# Personalized approach to primary and secondary prevention of ischemic stroke

**DOI:** 10.1186/1878-5085-5-9

**Published:** 2014-06-04

**Authors:** Jiří Polívka, Vladimír Rohan, Petr Ševčík, Jiří Polívka

**Affiliations:** 1 Department of Neurology, Faculty of Medicine in Pilsen, Charles University in Prague, Alej Svodody 80, Pilsen 304 60, Czech Republic; 2 Department of Neurology, University Hospital Pilsen, Alej Svodody 80, Pilsen 304 60, Czech Republic; 3 Department of Histology and Embryology, Faculty of Medicine in Pilsen, Charles University in Prague, Karlovarska 48, Pilsen 301 66, Czech Republic; 4 Biomedical Centre, Faculty of Medicine in Pilsen, Charles University in Prague, Karlovarska 48, Pilsen 301 66, Czech Republic

**Keywords:** Ischemic stroke, Primary prevention, Secondary prevention, Anticoagulation therapy, Antiplatelet therapy, Personalized medicine

## Abstract

Primary and secondary prevention of ischemic stroke represents a significant part of stroke management and health care. Although there are official guidelines concerning stroke management, new knowledge are introduced to them with a slight delay. This article provides an overview of current information on primary and secondary prevention of ischemic stroke. It summarizes information especially in the field of cardioembolic stroke, the use of new anticoagulants and the management of carotid stenosis based on the results of recent clinical studies. The optimal approach in stroke management is to follow these recommendations, to know new strategies and to apply an individual personalized approach in our clinical decisions.

## Review

### Introduction

Stroke is the third leading cause of death and the main cause of disability of adults in developed countries. Despite advances in prevention, the prevalence and incidence of ischemic stroke is expected to rise given the aging population [1]. A number of recommendations have been created on the management and prevention of stroke and transient ischemic attack. The guidelines of the European Stroke Organization (ESO) published in 2008 (updated in 2009) [2] cover stroke management in detail. These general recommendations should be transformed to individualized and personalized approach to each patient [3]. As there have been further advances since that time, this review provides an updated look at stroke management especially at stroke prevention.

### Primary prevention

Primary prevention aims to reduce the risk of ischemic stroke (IS) in subjects who have been asymptomatic and focuses on influencing and managing known risk factors such as arterial hypertension (AH), diabetes mellitus (DM) and disorders of lipid metabolism. The start and intensity of curative steps depends on an assessment of the total cardiovascular risk (CVR). In asymptomatic individuals, this value is determined by using nomograms from the Systematic Coronary Risk Evaluation (SCORE) [4] project, which evaluates the age, gender, systolic blood pressure (SBP), smoking habits and total cholesterol levels. A value over 5% is considered a high risk (probability of dying of cardiovascular disease in the next 10 years). Among symptomatic individuals with manifested cardiovascular disease, type 2 diabetes or type 1 diabetes with microalbuminuria, or chronic kidney disease, the risk is high (≥5%) or, if there are a combination of factors, very high (≥10%). The primary emphasis is placed on non-drug strategies and lifestyle changes—adopting a healthy diet with a higher proportion of fruits and vegetables and limited salt, increasing regular aerobic physical activity, reducing elevated body weight, limiting alcohol consumption and quitting smoking [2,4].

#### Arterial hypertension

For AH, which is a proven independent risk factor, the guidelines advocate correcting SBP to under 140 mmHg, except in older patients under 80 years of age, for whom there is a proven benefit of reducing SBP ≥160 to 150 − 140 mmHg. In patients over 80, each case must be assessed individually based on the subject's physical and mental condition. Also, the prehypertension (<120/80 mmHg) is associated with higher stroke morbidity [5]. The benefit of correcting SBP values under 140 mmHg has not been proven. A target diastolic value of under 90 mmHg is indicated; for diabetics, the target value is below 85 mmHg. In low-risk patients, non-drug strategies are primarily deployed first; if antihypertensive therapy is started, blood pressure values should be reduced only gradually. The choice of an antihypertensive agent depends on the patient's age and comorbidities, in older patients (over 80 years of age) calcium channel blockers or thiazide diuretics [6]. In women, the screening for arterial hypertension is indicated before prescribing oral contraceptives [7].

#### Diabetes mellitus

In DM patients, in addition to controlling blood glucose levels, greater emphasis is placed on controlling BP with a target value of under 140/80 mmHg. Angiotensin-converting enzyme inhibitors or angiotensin receptor antagonists are preferred for treatment [6]. Concomitant hypercholesterolemia should be corrected at low-density lipoprotein cholesterol (LDL-C) levels exceeding 3.0 mmol/L, primarily through statins [6,8].

#### Dyslipidemia

As another risk factor, dyslipidemia should be corrected in primary prevention with respect to the overall cardiovascular risk. Strategies should aim at influencing the LDL-C value by making lifestyle changes and, if necessary, through statin therapy [8-10] (Table 1).

**Table 1:** **Recommended target treatment levels for LDL-C (adjusted according to Catapano et al. **[8]**)**

**Cardiovascular risk**	**LDL-C target value**
Very high (manifest cardiovascular disease, type 2 DM, type 1 DM with organ impairment, moderate to severe kidney impairment or cardiovascular score ≥10%)	<1.8 mmol/L and/or ≥50% reduction of LDL-C
High (significantly increased individual risk factor, cardiovascular score 5%–10%)	<2.5 mmol/L
Moderate (cardiovascular score 1%–5%)	<3 mmol/L

#### Atrial fibrillation

The IS prevention guideline for patients with atrial fibrillation (AF) has undergone the most significant development in connection with the introduction of new oral anticoagulants (NOAs) and the availability of data from patients with implanted devices [11]. In patients with non-valvular AF, the stratification of IS risk has been re-evaluated to reflect the main and secondary clinically relevant risk factors when applying CHA_2_DS_2_-VASc (Table 2). Antithrombotic therapy is not recommended to AF patients over 65 years without additional risk factors, regardless of gender. As patients with severe renal insufficiency have not only a high risk of IS but also a high risk of death, heart attack and bleeding or hemorrhagic complications, they have been excluded from clinical studies. For this reason, it is difficult to evaluate the benefit of antithrombotic therapy, and it is not included in the score. The benefit of antithrombotic therapy in preventing IS must be higher than the risk of serious hemorrhage, especially intracerebral hemorrhage (ICH), the most feared complication of this type of therapy. It can be stratified using the HAS-BLED score (Table 3), which correlates well with the risk of ICH [12]. It was proven that in patients with the same HAS-BLED score who were treated with acetylsalicylic acid (ASA), the risk of ICH and major hemorrhagic complications is similar [13]. It is recommended to formally establish the risk of hemorrhage in all AF patients. Caution is necessary in case the HAS-BLED score is ≥3; however, this criterion does not exclude the patient from oral anticoagulant (OA) therapy, as the benefit of anticoagulant therapy exceeds the risk of hemorrhage even in patients with a high HAS-BLED score [14]. However, it is necessary to maximally compensate potentially reversible bleeding risk factors, such as uncontrolled arterial hypertension or the concomitant use of ASA or non-steroidal anti-inflammatory drugs (NSAID). In IS prevention among patients with non-valvular AF, ASA should be administered only to patients who reject any form of OA therapy [11].

**Table 2:** **CHA**_**2**_**DS**_**2**_**-VASc score (adjusted according to Lip et al. **[15])

**Risk factor**		**Score**
C	Congestive heart failure	1
H	Hypertension	1
A_2_	Age (≥75 years)	2
D	Diabetes mellitus	1
S_2_	Stroke (CS/TIA in history)	2
V	Vascular disease (myocardial infarction/peripheral vascular damage)	1
A	Age (65–74 years)	1
Sc	Sex category (female gender)	1

**Table 3:** **HAS-BLED score (modified according to Pisters et al. **[12])

**Risk factor**		**Score**
H	Hypertension (not controlled, >160 mmHg of systole)	1
A	Abnormal renal function or hepatic function	
	Transplant dialysis, Cr >200 μmol/L	1
	Cirrhosis, bilirubin >2× normal, AST/ALT/AP >3× normal	1
S	Stroke (CS in history, especially lacunar stroke)	1
B	Bleeding (bleeding in history or bleeding diathesis, anemia)	1
L	Labile INR (unstable or high INR)	1
E	Eldery (age ≥65 years)	1
D	Drugs/alcohol (antiplatelet medications, non-steroidal antiphlogistics or excessive use of alcohol)	1
		1

#### Other heart diseases

Anticoagulant therapy with warfarin in primary and secondary IS prevention is indicated in patients with a mechanical heart valve replacement (international normalized ratio (INR) 2.5–3.5), the presence of an intraventricular thrombus, mobile thrombus in the ascending aorta, dilated cardiomyopathy, especially in patients under 60 years of age [16], left atrial myxoma and/or mitral stenosis after a previous embolic event (INR 2–3).

#### New oral anticoagulants in the prevention of ischemic stroke

Until 2012, the only option for OA therapy for AF patients was vitamin K antagonists (VKA), mainly in the form of dose-adjusted warfarin with an INR of 2–3. Based on successful clinical studies which proved non-inferiority in comparison with warfarin in primary and secondary prevention of IS and peripheral embolization in patients with non-valvular AF—RE-LY [17], ROCKET-AF [18], ARISTOTLE [19] and ENGAGE-AF [20] — new oral anticoagulants (NOAs) were approved in 2012: first dabigatran as a direct thrombin inhibitor and later the direct Xa inhibitors rivaroxaban and apixaban, and edoxaban in 2013 in the USA. Of all NOAs that have been tested, clinical studies proved non-inferiority in comparison with warfarin, with better safety and reduced risk of ICH. This led to an update in the Guidelines for the Management of Atrial Fibrillation by the European Society of Cardiology [11], with NOAs considered more suitable for the majority of patients with non-valvular AF. Because there is only limited experience with NOAs, strict adherence to the approved indications and careful post-marketing monitoring are recommended. Given that there are no direct comparative studies among individual NOAs and indirect comparative analyses do not indicate that there are fundamental differences in efficacy, no conclusions can be drawn regarding preference for individual NOAs [21]. The advantage of NOAs over warfarin is their fixed dosage with no need for regular monitoring of anticoagulant activity. However, when determining the appropriate dosage, it is necessary to consider the patient's age and renal function. Another advantage is the lower quantity of clinically significant drug interactions. The short half-life and rapid onset and decrease in efficacy are important aspects that required careful compliance with treatment, as the anticoagulant effect is insufficient if more than one dose is skipped. Renal values must be monitored, especially for dabigatran. In polymorbid patients, these values may rapidly change in the course of an intercurrent disease [22]. Unlike warfarin, no haemocoagulation test can be used for NOAs that would clearly quantify the anticoagulation effect. Non-specific anticoagulation tests may be used, such as activated partial thromboplastin time (APTT); thrombin time or ecarin clotting time tests are more specific for dabigatran, plus prothrombin time (PT) or determining anti-Xa activity for Xa inhibitors. However, these tests are used more to determine the presence of a medication and cannot be reliably used to estimate the anticoagulant effect of the NOA. As yet, no NOAs have a specific antidote. To rapidly adjust coagulation in case of serious bleeding, in addition to blood derivatives, specific procoagulant reversal agents such as prothrombin complex concentrate (PCC), activated prothrombin complex concentrate (APCC) or recombinant factor VIIa (r-FVIIa) may be administered. Hemodialysis may also be considered for dabigatran, but practical experience is still limited [23].

#### Asymptomatic stenosis of the internal carotid artery

Atherosclerotic stenosis of the extracranial part of the internal carotid artery (ICA) is associated with an increased risk of IS [24]. The risk of asymptomatic stenosis progression rises over time, depending on the presence of additional risk factors such as smoking, arterial hypertension, DM, the level of stenosis, the composition of the plaque and contralateral impairment of the ICA [25]. In short-term monitoring, the risk of ipsilateral IS in patients with asymptomatic ICA stenosis ranges between 1% and 3%, depending on the seriousness of the stenosis and the studied population [24]. As there have been significant advances in conservative and invasive treatment, no valid data is available right now that assesses the real risk of asymptomatic ICA stenosis comparing these two different management strategies. In a 10-year study monitoring patients in the ACST trial [26], CEA reduced the risk of stroke, including perioperative stroke, to 13.4%, compared to 17.9% in patients with delayed intervention or conservative treatment. The fact that 80% of the patients in the study were not treated with statins may influence an interpretation of the results. In regard to a comparison of carotid endarterectomy (CEA) and carotid artery stenting (CAS), the last extensive study was CREST [27], which compared these two strategies in symptomatic and asymptomatic patients with significant ICA stenosis. During a mid-term 2.5-year monitoring study, the 4-year risk composite target (IS, myocardial infarction or death) was found to be nearly the same for both CAS and CEA (7.2% and 6.8%), regardless of age or clinical presentation of the stenosis. Surprisingly, patients over 70 years of age profited more from CEA, while patients under 70 profited more from CAS. The risk of CS or death was 6.4% for CAS and 4.7% for CEA, with the difference nearing significance only among asymptomatic patients. Only differences in perioperative complications were significant, with a higher risk of CD in CAS (4.1% vs. 2.3%) and a higher risk of myocardial infarction in CEA (1.1% vs. 2.3%). The risk was similar in the subsequent period (2.0% vs. 2.4%). The results of this study do not fundamentally change the current guideline that in the case of significant asymptomatic ICA stenosis (60%–90% according to the North American Symptomatic Carotid Endarterectomy Trial—NASCET), intensive drug treatment of the risk factors (DM, arterial hypertension, dyslipidemia) is indicated. CEA is indicated only among patients with a high risk of stroke (men, stenosis >80%, plaque character) and expected survival greater than 5 years, performed in centres where the operative risk is less than 3%. Administration of ASA is indicated before and after CEA. CAS is not indicated for asymptomatic subjects.

### Secondary prevention

Secondary prevention aimed at reducing the risk of another IS must be based on the etiology of the past ischemic IS and consider the presence of any additional risk factors. It consists of the optimal compensation of vascular risk factors—arterial hypertension, hyperlipidaemia and diabetes, antiplatelet or anticoagulant therapy and, in indicated cases, the use of invasive surgical or endovascular therapy. The patient's regimen, emphasizing adequate physical activity, elevated body weight loss, sufficient hydration, dietary changes, quitting smoking and a reduction in excessive alcohol consumption, is an integral part of this [2].

#### Vascular risk factors

As part of optimizing vascular risk factors, after the acute stroke phase subsides, antihypertensive therapy that adjusts BP to normal values is indicated. The BP value must be individualized with regard to possible haemodynamic consequences, however, such as in patients with bilateral stenosis in afferent cerebral arteries or the trunk of the cerebral artery. In contrast, in patients with small artery damage, reducing pressure below 130 mmHg SBP [28] seems suitable. As in primary prevention, individualized DM therapy is indicated. In non-cardiogenic stroke, statin therapy with a target LDL value under 2.5 among high-risk subjects under <1.81 mmol/L is indicated [9]. In patients with sleep-disordered breathing, respiratory therapy with continual positive pressure in the air passages is recommended [29].

#### Atherothrombotic stroke

In most cases, antiplatelet therapy is indicated for secondary prevention of atherothrombotic (non-cardioembolic) stroke. According to the ESO guidelines, ASA should be administered in combination with dipyridamole (25/200 mg 2× daily) or clopidogrel monotherapy (75 mg/day), alternatively, as an economical alternative ASA monotherapy (75–325 mg/day). The efficacy of higher doses of ASA has not been proven [30]. The combination of ASA + clopidogrel versus clopidogrel monotherapy and the combination of ASA + clopidogrel versus ASA monotherapy have been investigated in MATCH [31] and CHARISMA [32], respectively. In both studies, with a long-term administration of ASA + clopidogrel, the insignificant reduction in the risk of ischemic stroke was accompanied by a significant increase in bleeding complications and mortality. The last SPS3 trial of lacunar stroke came to the same conclusion [33]. The coincidence of stroke and a recent myocardial infarction or status post coronary stenting thus remain the indications for using a combination of ASA + clopidogrel. The new specific indication for the combination of short-term ASA + clopidogrel therapy seems to be significant intracranial symptomatic stenosis in a major artery. The SAMMPRIS study investigated the effect of intracranial stenting and intensive drug therapy (ASA + clopidogrel + statin) versus intensive drug therapy alone [34]. It showed a significantly higher incidence of stroke and death in the stented group during the 30-day (14.7% vs. 5.8%) and the 1-year (20.0% vs. 12.2%) monitoring period. At the same time, the study showed about a 50% drop in the incidence of IS and death in the medicated group compared to historical controls—patients treated with ASA or warfarin in the WASID study [34,35]. In the case of stroke, it is also necessary to consider possible resistance to ASA or clopidogrel when starting antiaggregant therapy. It is necessary to evaluate the compensation of other vascular risk factors, especially the etiology of the stroke with regard to the potential for cardioembolism, especially in the case of paroxysmal AF.

#### Cardioembolic stroke

According to the ESO guidelines, anticoagulant therapy with warfarin (INR 2–3) is indicated in the secondary prevention of IS in patients with AF both paroxysmal and permanent and also in other cardioembolism or NOAs in the case of AF. Regarding adherence in the management of atrial fibrillation, there are the problems of high differences in the management and compliance [36]. In cryptogenic stroke, paroxysmal atrial fibrillation should always be excluded. Its detection rate is dependent on the intensity of ECG monitoring [37,38]. When deciding on the timing of starting full anticoagulant therapy, one must consider the risk of the hemorrhagic transformation of infarction foci with regard to size and location. The benefit of early anticoagulant therapy versus a delayed start has not been proven [39]. Secondary prevention with ASA alone has little effect, and the risk of major bleeding is not significantly different from OA [13]. ASA + clopidogrel compared to warfarin in patients with AF also shows low efficacy and does not bring a significant reduction in the risk of bleeding complications—ACTIVE W study [40]. Antiplatelet therapy should be restricted to patients who reject any form of OA therapy. The NOAs discussed above are an alternative to warfarin.

In the case of concurrent acute myocardial infarction, concurrent anticoagulant and antiaggregant therapy for a 3-month period is indicated with regard to the size of the infarction foci and the risk of hemorrhagic transformation. As in primary prevention, anticoagulant therapy with warfarin is also indicated in the case of other cardiac sources of embolism.

#### Patent foramen ovale

The significance of patent foramen ovale (PFO) is still discussed and studied in patients with cryptogenic stroke. The results of three randomized studies were published in 2012—CLOSURE I [41], PC-Trial [42] and RESPECT [43], comparing the effect of PFO closure with ASA or warfarin drug treatment. REDUCE, a study comparing PFO closure with antiplatelet treatment vs. antiplatelet treatment as monotherapy, is ongoing [44]. Given the low incidence of target events (stroke, death) and relatively short period of monitoring (2 years), none of the studies have shown a statistically significant difference among the monitored groups, despite a certain trend towards mechanical closure of PFO. Subanalyses and meta-analyses of these studies may provide further data. In contrast, data from observational studies with a longer period of monitoring report a statistically significant difference in favor of invasive vs. drug therapy, and in the case of drug therapy, there is a significant benefit of anticoagulant therapy vs. antiplatelet therapy [45]. Although for the reasons described above there is still not enough clear clinical evidence, it is appropriate to consider PFO closure only in patients with embolic-type stroke with a significant shunt in the transesophageal echocardiogram exam and if other risk factors are absent. In other cases, anticoagulant or antiplatelet therapy is indicated.

#### Thrombophilia

In patients with ischemic stroke of unclear etiology or who are under 40 years of age, testing for thrombophilia is indicated. Anticoagulant therapy is normally indicated in the case of a proven deficiency of antithrombin III, protein C and protein S; resistance to activated protein C (factor V Leiden), especially in the case of deep vein thrombosis, is also detected. Patients with positive antiphospholipid antibodies with no other signs of antiphospholipid syndrome are indicated to only take antiplatelet therapy; anticoagulant therapy is indicated for patients meeting the criteria for antiphospholipid syndrome [46].

#### Major extracranial arterial stenosis

As is the case for other atherothrombotic IS, intensive drug treatment of the risk factors and antiplatelet therapy are indicated for secondary prevention in patients with major extracranial arterial stenosis [2,46]. In the question of using CEA or CAS in patients with significant ICA stenosis, there have not been any fundamental changes to the ESO guidelines; there is still a lack of data comparing these strategies with current intensive drug therapy. Early CEA within 2 weeks after the stroke is indicated for patients with small-scale infarction who do not have a high risk of hyperperfusion syndrome with potential hemorrhagic transformation of the infarction foci. The benefit of CEA at an interval of 3 months is minimal compared to conservative treatment. In addition to the seriousness of the stenosis, the characteristics of the plaque also plays a role in indicating CEA/CAS, with the presence of ulcers being an indication to operate even lower-level stenosis (50%–69%) while adhering to the principles of low perioperative morbidity and mortality (<3%). Even in the perioperative period, patients should be left on antiplatelet therapy. CAS is recommended only for patients in whom CEA is contraindicated, the location of the stenosis is not surgically accessible, with restenosis following CEA and post-radiation stenosis. After CAS, dual antiplatelet therapy with ASA + clopidogrel for a period of 1 month is indicated.

#### Intracranial arterial stenosis

Dual antiaggregant therapy with ASA and clopidogrel in combination with optimal compensation of vascular risk factors was compared against the effect of stenting symptomatic intracranial large artery stenosis (50%–99%) in the SAMMPRIS study [34]. Enrolment in the study was halted after 451 patients were included in the study, due to a significantly higher incidence of early stroke/death after stenting compared to conservative treatment (14.7% vs. 5.8%).

## Conclusions

Stroke management is a great challenge. Stroke prevention remains the object of intense medical research. Based on new information provided in this review, management strategies of stroke prevention should be personalized, even if general guidelines exist. The management of arterial hypertension, diabetes, dyslipidemia, atrial fibrillation and the other causes of cardioembolic stroke in the primary and secondary stroke prevention, search for thrombophilia in younger patients and the management of extracranial arterial stenosis and the indication and use of new oral anticoagulants are of cardinal importance. An update to the current guidelines can also be expected.

## Abbreviations

AF: atrial fibrillation; AH: arterial hypertension; APCC: activated prothrombin complex concentrate; APTT: activated partial thromboplastin time; ASA: acetylsalicylic acid; CAS: carotid artery stenting; CEA: carotid endarterectomy; DM: diabetes mellitus; ESO: European Stroke Organization; ICH: intracerebral hemorrhage; INR: international normalized ratio; IS: ischemic stroke; NASCET: North American Symptomatic Carotid Endarterectomy Trial; NOAs: new oral anticoagulants; PCC: prothrombin complex concentrate; PFO: patent foramen ovale; PT: prothrombin time; r-FVIIa: recombinant factor VIIa; SBP: systolic blood pressure; TIA: transient ischemic attack.

## Competing interests

The authors declare that they have no competing interests.

## Authors’ contributions

PJ and RV conceived the review and coordinated the drafting of the manuscript. SP and PJ Jr. participated in the design of the review, performed literature searches and identified relevant studies. PJ and RV provided content expertise. All authors read and approved the final manuscript.
